# Exploring the rate-limiting steps in visual phototransduction recovery by bottom-up kinetic modeling

**DOI:** 10.1186/1478-811X-11-36

**Published:** 2013-05-21

**Authors:** Brandon M Invergo, Ludovica Montanucci, Karl-Wilhelm Koch, Jaume Bertranpetit, Daniele Dell’Orco

**Affiliations:** 1 IBE – Institute of Evolutionary Biology (CSIC-Universitat Pompeu Fabra), CEXS-UPF-PRBB, Barcelona, Catalonia, Spain; 2 Department of Neurosciences, Biochemistry Group, University of Oldenburg, Oldenburg, Germany; 3 Department of Life Sciences and Reproduction, Section of Biological Chemistry and Center for BioMedical Computing (CBMC), University of Verona, Strada le Grazie 8, 37134, Verona, Italy

**Keywords:** Visual phototransduction, G-protein-coupled receptor signaling, Arrestin, Recoverin, Kinetic modeling, Systems biology

## Abstract

**Background:**

Phototransduction in vertebrate photoreceptor cells represents a paradigm of signaling pathways mediated by G-protein-coupled receptors (GPCRs), which share common modules linking the initiation of the cascade to the final response of the cell. In this work, we focused on the recovery phase of the visual photoresponse, which is comprised of several interacting mechanisms.

**Results:**

We employed current biochemical knowledge to investigate the response mechanisms of a comprehensive model of the visual phototransduction pathway. In particular, we have improved the model by implementing a more detailed representation of the recoverin (Rec)-mediated calcium feedback on rhodopsin kinase and including a dynamic arrestin (Arr) oligomerization mechanism. The model was successfully employed to investigate the rate limiting steps in the recovery of the rod photoreceptor cell after illumination. Simulation of experimental conditions in which the expression levels of rhodospin kinase (RK), of the regulator of the G-protein signaling (RGS), of Arr and of Rec were altered individually or in combination revealed severe kinetic constraints to the dynamics of the overall network.

**Conclusions:**

Our simulations confirm that RGS-mediated effector shutdown is the rate-limiting step in the recovery of the photoreceptor and show that the dynamic formation and dissociation of Arr homodimers and homotetramers at different light intensities significantly affect the timing of rhodopsin shutdown. The transition of Arr from its oligomeric storage forms to its monomeric form serves to temper its availability in the functional state. Our results may explain the puzzling evidence that overexpressing RK does not influence the saturation time of rod cells at bright light stimuli. The approach presented here could be extended to the study of other GPCR signaling pathways.

## Background

Phototransduction is the biochemical process by which a visual stimulus initiates a neuronal response in the photoreceptor cells of the retina. The capture of a photon by a visual pigment molecule triggers a G-protein-coupled receptor signaling cascade that leads to the closure of cGMP-gated ion channels, which decreases intracellular Ca^2+^ and causes the hyper-polarization of the cell membrane. Changing Ca^2+^ concentrations in the 0.1 – 0.6 μM range contribute to at least three feedback processes, which serve to regulate tightly the recovery of a photoresponse [1]. One particular means of Ca^2+^ feedback is exerted through recoverin (Rec), a protein which binds and regulates the action of rhodopsin kinase (RK). When Rec binds two Ca^2+^ ions, it undergoes a conformational change, exposing a myristoyl group [2]. This “relaxed” (RecR_Ca_) form prevails in the dark, at higher intracellular Ca^2+^ concentrations, and increases the protein’s affinity for the disk membrane due to an overall augmented hydrophobicity [3]. In the relaxed form, Rec binds RK and prevents it from phosphorylating the activated photopigment rhodopsin (R*) [4-9]. As Ca^2+^ concentration decreases during a photoresponse, Rec reverts to its tense form (RecT), becoming more hydrophilic, and ceasing to bind RK. The free RK may then phosphorylate R*, leading to the deactivation of R* by arrestin (Arr) and the termination of the response.

One mechanism of the phototransduction cascade whose function remains unclear is the formation of homo-oligomers by Arr. It is known that in dark-adapted rod cells, Arr exists in a steady-state equilibrium of monomers, dimers and tetramers and that during light-adaptation the oligomeric forms dissociate, increasing the concentration of monomeric Arr available to quench R* [10-12]. It is generally thought that the dimers and tetramers together function as storage forms and exist to maintain the ideal concentration of available, monomeric Arr, however the kinetic implications of Arr oligomerization have not been fully explored. Supplementary to biochemical and transgenic animal assays, detailed modeling of the phototransduction system could provide key insights into the dynamic effects of this and other mechanisms.

Mathematical simulations of biochemical networks are essential for gaining a proper understanding of the complex dynamics underlying them. As perhaps one of the best-understood GPCR-mediated signaling pathways, the phototransduction system has seen a rich history of modeling. This has led to comprehensive models of the amphibian phototransduction cascade, which include a large portion of the known reactions that occur during phototransduction [1,13-15]. Despite the intrinsic limits brought in by the well-stirred approximation, such comprehensive models have proven realistic and widely useful. For instance, systems-level analysis of the dynamics underlying phototransduction has been instrumental in predicting novel functions of signaling proteins in the network including rhodopsin (ref. [16]) and in unraveling some molecular mechanisms associated to disease (ref. [15]). Different modeling approaches account for spatial dynamics of the processes occurring in specific cell compartments [17,18]. While they provide better physical description of the processes, of great relevance to single photon response dynamics, to date these approaches have been applied to limited subsets of reactions.

Recently, a systems biology approach was used to build a comprehensive model of phototransduction in rod cells based on most of the known biochemical information on the signaling processes [15]. The model relied on the quantitative data collected over 40 years of biochemical and biophysical research on amphibian rod photoreceptors, and included all of the modular components of the signaling cascade, namely: a) the activation of the receptor rhodopsin by light stimuli of different duration and intensity; b) the signal amplification steps occurring upon transferring the information from the receptor to the G protein (transducin) and from the G protein to the effector phosphodiesterase 6 (PDE); c) the Ca^2+^ −mediated feedback mechanisms on the regulation of processes occurring both on discs and on the plasma membrane; and d) the deactivation of both receptor and effector for normal recovery of dark-adaptation cell conditions.

The model successfully reproduced a number of experimental data collected on photoreceptors from different species, stimulated by light ranging over five order of magnitude in intensity [15,19]. Interestingly, while the quantitative comparison between simulations and experimental data showed striking consistency for amphibian cells, the model proved also able to predict with great accuracy the qualitative dynamics observed in mouse photoreceptors [15]. This latter finding proved the great potential of the system-level analysis, which apparently is able to capture the evolutionarily important topological and dynamic constraints in the same signal transduction pathways in different species. Moreover, the model reproduced and predicted dynamic behaviors observed with the widely used approach of producing transgenic mice with genetic manipulations aimed at investigating the specific role of genes and their products in regulating the cascade. The development of this model has resulted in a framework in which the phototransduction process is broken down into its fundamental reactions, which are then modeled largely according to the law of mass action. This resulting model is effectively modular in design, allowing relatively isolated modifications to a mechanism without great disruptions to the others [20,21].

We have taken advantage of this modular structure in order to incorporate the latest knowledge on the kinetics of Ca^2+^-mediated Rec feedback on R* shutdown into a new iteration of this model. We then further extended it to include a dynamic Arr oligomerization mechanism. The modified model provides an unprecedented ability to probe this mechanism’s influence on the dynamics of R* shutdown. Simulations performed with the present model allowed full reproducibility of a number of diverse experimental results, including experiments performed with photoreceptors from animals carrying genetic modifications in the key components of the shutdown machinery.

Using the present model, we have replicated several published experiments, not only to verify that the model can accurately reproduce the experimental results but also to summarize and explore the current knowledge of phototransduction recovery mechanisms and the contributions of these two mechanisms to the process. We found that the model gives qualitatively accurate reproduction of experimental data (accounting for species differences between the amphibian model and the experiments, which were largely performed on mice). In particular, the results suggest an important modulatory role for the process of Arr oligomerization. Lastly, we note that to perform such extensive experimentation with animals in a laboratory would be extremely expensive and time-consuming, pointing to a beneficial role of large-scale system modeling in the experimentation process.

## Results

### A comprehensive, system-level description of the signaling cascade

We have improved and extended the model of Dell’Orco *et al*. (ref [15]), making significant steps forward in the comprehensive, biochemistry-based description of the phototransduction cascade. We herein focused on the still largely unclear mechanisms constituting rate-limiting steps in the kinetics of cell recovery after illumination. First, we reconsidered the affinity relationships of RK and Arr with phosphorylated R*. Previously, these affinities varied exponentially with the number of phosphates attached to R*. Based on experimental data and considerations for goodness-of-fit versus parameter sensitivity, we replaced RK’s exponential decrease in affinity and Arr’s exponential increase in affinity with respective linear relationships (see Methods).

**Figure 1:**
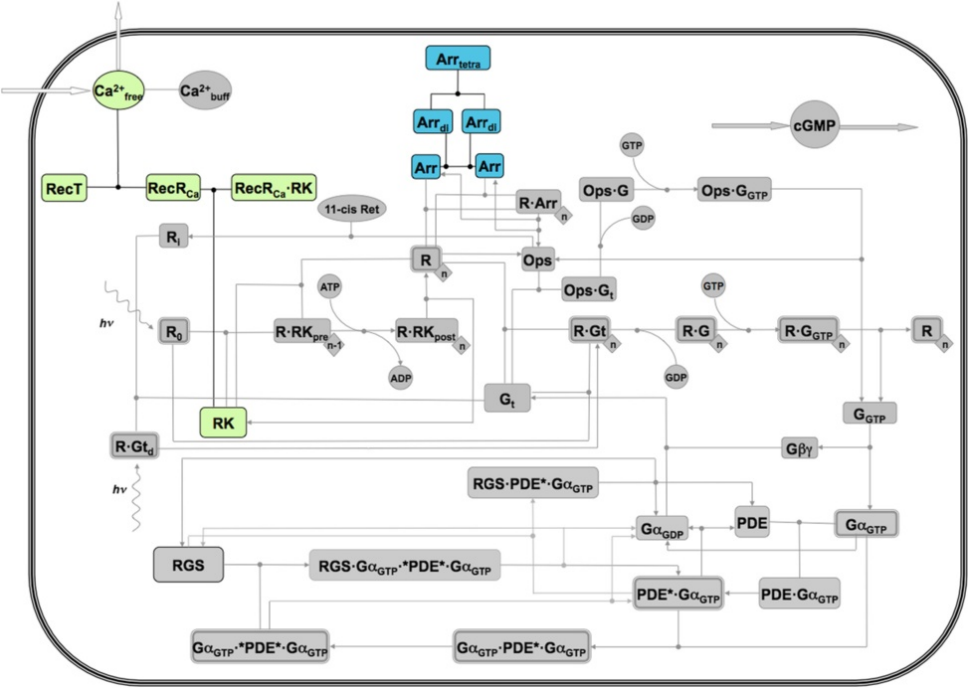
**Network structure of the extended model of vertebrate phototransduction.** With respect to previous implementations (refs. [15,19]), the new model accounts for more realistic molecular steps involved in the recovery after light activation. In particular, a detailed mechanism of Rec-mediated calcium feedback on RK was added (light green boxes) together with dynamic Arr oligomerization, leading to the formation of Arr dimers and tetramers (blue boxes). The biochemical reactions describing these new modules and the relative kinetic parameters are described in the Methods. Irreversible reactions are marked with an arrow indicating the direction of each reaction. The symbols are the same used in ref. [15].

We next replaced a representation of the Rec-mediated Ca^2+^ feedback on RK based on a quasi-steady state assumption with a realistic process consisting of three fundamental reactions. This permitted the removal of another exponential parameter (*w* in the model of Dell’Orco *et al*. (ref [15])) as well as the explicit modeling of Rec as a significant Ca^2+^ buffer. Finally, we implemented a dynamic Arr homo-oligomerization mechanism, in which Arr self-associates to form homodimers and homotetramers. The implementation of the Rec-mediated calcium feedback on RK and the dynamic Arr oligomerization mechanism was realized as illustrated in the Methods session. The new model thus resulted in an extended network of interactions (Figure 1), which we used to explore, by numerical simulation, currently inaccessible molecular scenarios, in which the expression level of key proteins were varied in order to gain insights into the regulatory mechanisms acting in the recovery phase of the phototransduction cascade.

#### Model validation

**Figure 2:**
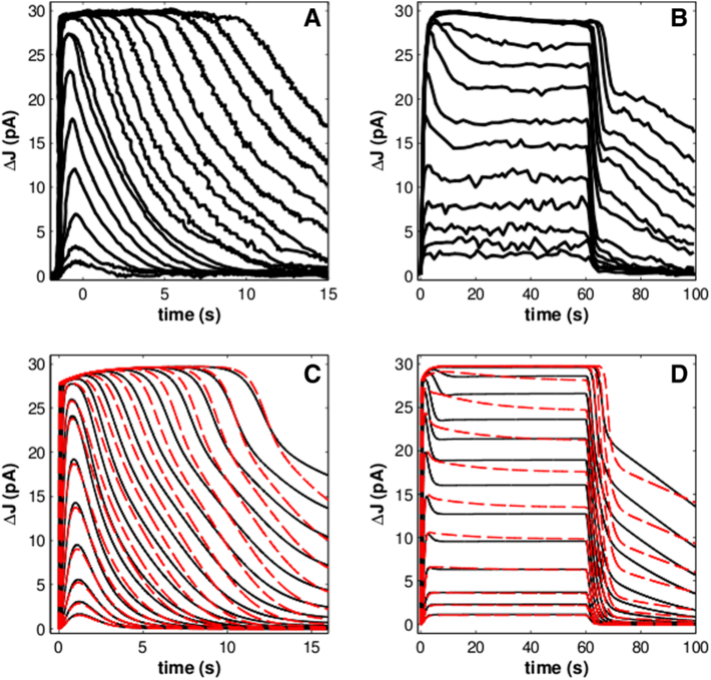
**A comparison between experimental data (A, B) and simulated responses (C, D).** (**A**, **C**): Flash responses generated by a dark-adapted toad rod over 5 orders of magnitude in light stimulus as a 24 ms flash, ranging in intensity from 1.5 R* per flash to 118,000 R* per flash. (**B**, **D**): Responses from a newt rod for 60 s steps of light ranging from 5 R* to 220,000 R*. Black traces are from the model of Dell’Orco & Koch (2011; ref. [19]); red dashed traces are from the present model. The two models do not vary greatly from each other for flash responses. When subjected to steps of light, the present model saturates at lower stimuli than the previous models and, in this regard, is more similar to the experimental data.

We verified the present model by recapitulating simulation results published previously by Dell’Orco *et al*. [15], in which illumination intensities extended over five orders of magnitude. The new model features were found not to alter greatly the system’s dynamics for a wide range of experimental conditions. Responses to series of brief flashes, leading from few rhodopsin photoisomerizations up to saturating levels, or to series of prolonged pulses of light of increasing intensity both closely matched experimental data and previously simulated results (Figure 2A & B). Notably, when exposed to prolonged pulses (60s) of light of stronger intensity, this model shows decreased time-to-plateau recovery after the rising phase at all light intensities. Indeed, inspection of the experimental data reveals a similar pattern, with slower recovery transients preceding the plateau [22].

The performance of the modified model in reproducing light adaptation rather than dark-adapted behaviors was also checked (Additional file 1: Figure S1). Both reduced light sensitivity and accelerated recovery when a saturating stimulus is delivered in the presence of a non-saturating background of light were accurately reproduced by the model (Additional file 1: Figure S1).

**Figure 3:**
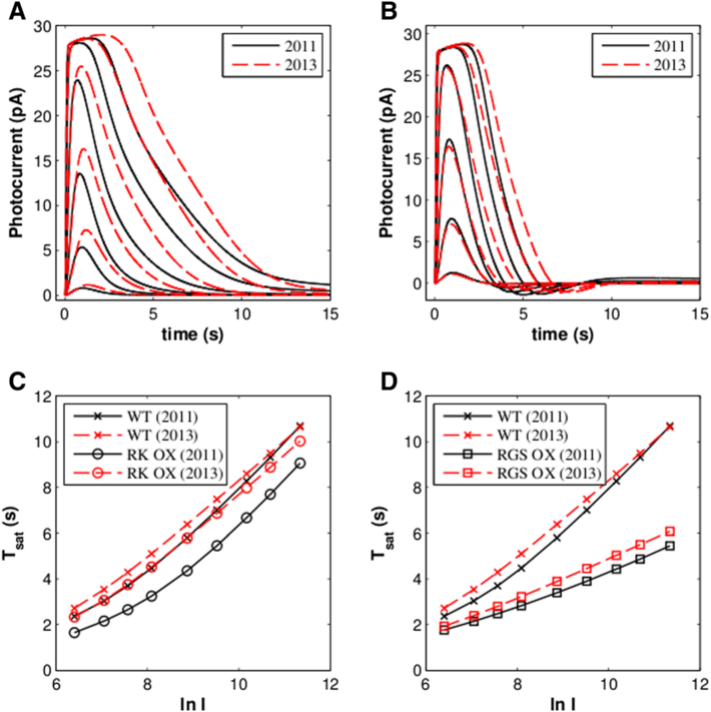
**Simulated responses under mutant conditions: 2.4x RK overexpression (A, C) and 2.3x RGS overexpression (B, D).** Panels **A** and **B** present flash responses while **C** and **D** show the Pepperberg plots for saturating flashes (X’s: WT; open circles: RK overexpression; open squares: RGS overexpression). RGS expression results in greatly accelerated recovery, manifested in both decreased saturation time and reduced *τ*_*D*_, the slope of a regression line fitting the first four points. RK overexpression results in strongly decreased saturation times only for the model of Dell’Orco & Koch (2011; ref. [19]). This effect is attenuated in the present model.

The modified model was also tested in its capability of reproducing photoresponses by genetically modified animals (See Methods and Additional file 1). Simulations generally reproduced the experimental data well. However, unlike the previous implementation of the dynamic model, the simulation of RK overexpression more accurately reproduced experimental results [15]. Indeed, in the simulated results of Dell’Orco *et al*. [15], RK overexpression resulted in shorter saturation times (*T*_*sat*_; the duration of time that a response remains at at least 90% of the maximal amplitude after a saturating flash stimulus) after bright flashes (Figure 3), which differs from experimental results; it has previously been shown that *T*_*sat*_ of rods overexpressing RK is not significantly different than that of wild-type (WT) rods [23]. The new model largely resolves this discrepancy.

The model was then employed to elucidate the complex deactivation dynamics of phototransduction. Many experiments have been performed previously to explore these processes through the use of transgenic animals or other means of altering the expression levels of key proteins to determine their roles. Using the present model, we recapitulated several of these experiments. Because much current research on the phototransduction system is performed using transgenic mice, we were precluded from performing quantitative comparisons against the amphibian-based model. However, as previously demonstrated by Dell’Orco *et al*. [15], the model with its set of kinetic constraints is capable of generating results that are qualitatively comparable to experimental data, despite species differences, across a wide range of experimental conditions (Figures 2, 3 and Additional file 1: Figure S2-S3).

We will now present in detail the most salient features of the simulated photoresponses obtained with the extended model and make direct comparisons with the known experimental data.

### Increased RK availability accelerates recovery from saturation

**Figure 4:**
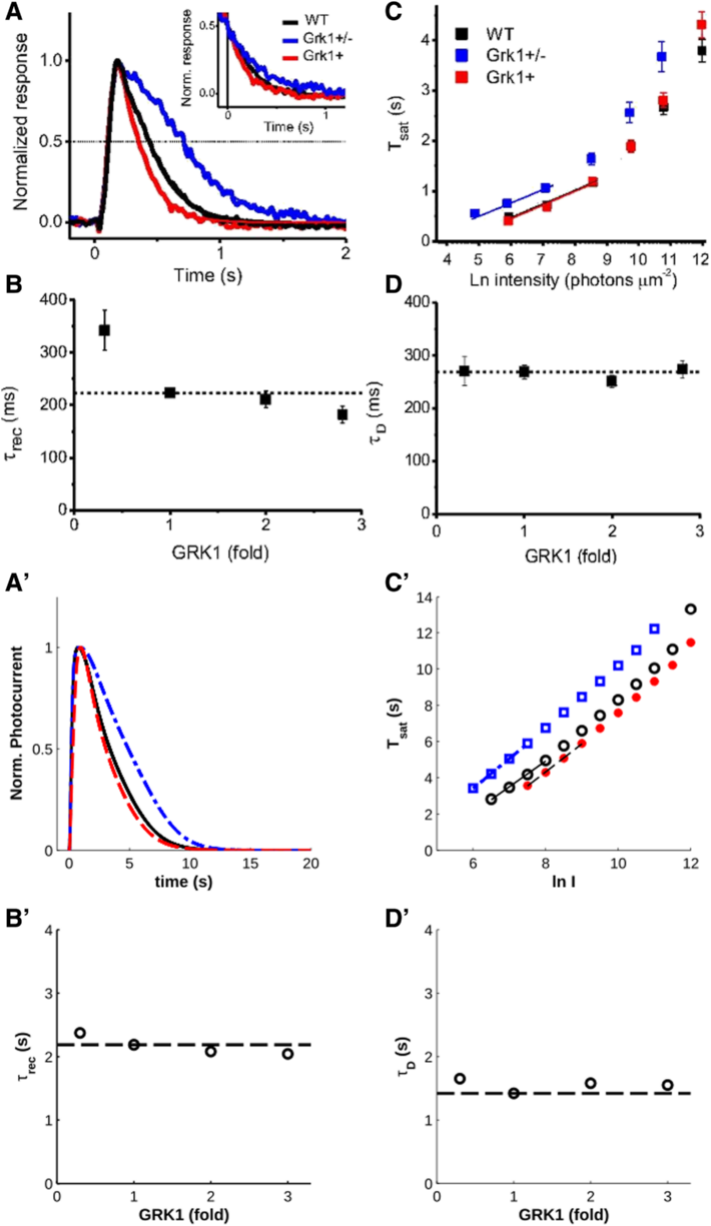
**The effects of varying RK (GRK1) expression on the photoresponse, as carried out by Sakurai et al. (ref. **[24]**. **(**A′-D′**) Simulations of the same experiments. **A′**) Normalized responses to a non-saturating flash (135 R*). 0.3x RK underexpression (dotted-dashed traces) leads to a slowed normalized recovery to a non-stimulating flash, while recovery is slightly accelerated for 3x RK overexpression (dashed traces). **B′**) The time constant of recovery, τ_rec_, as a function of RK expression. **C′**) Typical Pepperberg plots (ref. [25]) reporting the saturation time, T_sat_, as a function of stimulus intensity (in log units; WT: circles; 0.3x RK underexpression: squares; 3x RK overexpression: stars). 0.3x RK underexpression results in higher than normal T_sat_ values for saturating flashes. 3x RK overexpression, on the other hand, results in a slight decrease in T_sat_. **D′**) The dominant time constant of recovery from saturating flashes, τ_D_, is not affected by RK expression levels. (Panels **A**-**D** copyright © 2011 The Association for Research in Vision and Ophthalmology, Inc.). NB: the authors used “Grk1” and “GRK1” interchangeably, and used “GRK1+” to indicate overexpression instead of the more common “GRK1+/+”).

RK plays a major role in R* shutdown by regulating its phosphorylation, which finally determines the capping by Arr, therefore it is not surprising that much experimental effort in recent years has been made to elucidate the quantitative effects of alterations in the expression level of RK on the overall cascade. In this respect, in a recent work Sakurai *et al*. generated lines of transgenic mice with different expression levels of RK (two-and three-fold overexpression compared to the wild type) [24]. For mice overexpressing RK, they found results that conflicted with those of Krispel *et al*. (ref. [23]), who had produced similar transgenic mice and found that RK overexpression had neither an effect on *T*_*sat*_ for saturating flashes nor on the recovery time constant (*τ*_*rec*_; the time constant of a single exponential function fit to the second half of the recovery phase of a non-saturating response). Sakurai *et al*. found that, for their animals, while the two- and three-fold overexpression of RK did not have an effect on *T*_*sat*_, in agreement with the previous findings, it did, in fact, lead to a reduction in *τ*_*rec*_ for non-saturating responses. When we simulated their experiments, we found the same pattern of a decrease in *τ*_*rec*_ with increasing RK quantity (Figure 4A′ & B′). When the model was tested with saturating flashes, the model showed a slight decrease in *T*_*sat*_ with RK overexpression compared to wild-type (WT) for similar flash intensities, in contrast to the experimental results (Figure 4C′).

Furthermore, Sakurai *et al*. found that the dominant time constant of recovery from a saturating response (τ_D;_ measured as the slope of *T*_*sat*_ over logarithmically increasing stimulus intensities, visualized in a so-called Pepperberg plot as in Figure 4B and B′ [25]), did not significantly change with RK expression levels. The interpretation given by the authors for this result is that the measurement of *τ*_*D*_ occurs after RK-mediated rhodopsin (R*) shutoff has been completed and thus only reflects the rate-limiting step of RGS-mediated effector shutdown. The measurement of *τ*_*rec*_, on the other hand, may still be influenced by the rate of R* shutoff [24,26] This stands in contrast to the results of Krispel *et al*., who found that *τ*_*D*_ slightly increased with RK two-fold overexpression [23]. In simulations, we found that *τ*_*D*_ did not vary greatly with RK expression (Figure 4D, Table 1). Since *τ*_*D*_ is relatively constant with increasing RK expression, the rate-limiting step of recovery is unaffected by RK activity in the model.

**Table 1:** ***τ***_***D***_** Values for simulated experiments**

**Experiment**	***τ***_***D***_	**Normalized** ***τ***_***D***_
WT	1.42	1
0.3x RK UX	1.65	1.16
0.4x RK UX	1.53	1.08
2x RK OX	1.58	1.11
3x RK OX	1.55	1.09
Rec KO	1.38	0.97
4x Rec OX	1.73	1.22
0.2x RGS UX	6.86	4.83
2x RGS OX	0.83	0.58
4x RGS OX	0.60	0.42
6x RGS OX	0.53	0.37
0.4x RK/6x RGS	0.69	0.49
3x RK/0.2x RGS	7.30	5.14

τ_D_ values, or the change in saturation time with logarithmically increasing stimulus intensities, for the various experiments simulated herein. Normalization is done against the wild-type value. (WT = wild-type, UX = underexpression, OX = overexpression, KO = knock-out). Only RGS mutants have significant effects on τ_D_.

**Figure 5:**
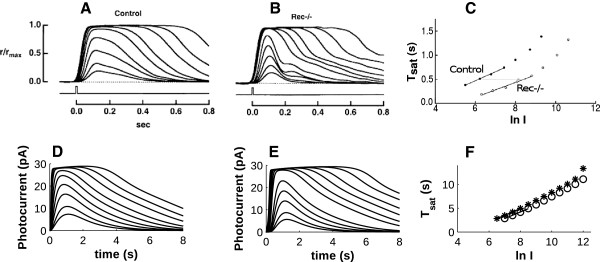
**Experimental (A-C; ref **[27]**) and simulated (D-F) flash responses (A, B, D, E) and Pepperberg plots (C, F) showing reduced sensitivity and shorter saturation times in animals lacking Rec (B, E) compared to wild-type (WT) (A, D).** Note the difference in time scale is due to species differences. Simulated stimulus intensities were adapted from Makino et al. [27] (see Methods). Simulated WT stimuli were 9.44, 16.48, 34.24, 59.92, 111.2, 194.4, 403.2, 705.6 and 1352 R* per flash (**D**). Simulated Rec−/− stimuli were 10.24, 17.92, 37.2, 65.12, 120.8, 438.4, 768, 1472, 2584, 5360 and 9360 R* per flash (**E**). Eliminating Rec activity results in only a slight decrease in saturation time across a range of saturating stimulus intensities compared to the significant shift seen in the experimental results (**C**, **F**; open circles: WT; stars: Rec−/−). (Panels **A**-**C** copyright © 2004 Makino et al.).

Intuitively, an overabundance of available RK due to the complete absence of Rec inhibition acting on it would be expected to show similar dynamic behavior to an overabundance of RK due to its overexpression. However, experiments using Rec knock-out mice prove otherwise [27]. When Rec is absent, phototransduction signaling shows decreased sensitivity and shorter saturation times (Figure 5A & B). Unlike the case of RK overexpression, Rec knockout animals not only have shorter *T*_*sat*_ than WT for similar stimulus intensities, but they also show a slight reduction in *τ*_*D*_. Moreover, the shape of the decreasing phase of the photoresponses appears to differ from that of the control (compare Figure 5A and B), as in the case of Rec−/− three phases are clearly visible at each light intensity (Figure 5B). When these conditions were simulated, the decreasing photoresponse remained substantially biphasic, moreover we saw only a moderate shift in *T*_*sat*_, similar to the result of RK overexpression, and no significant change in *τ*_*D*_ (Figure 5C; Table 1). Thus, whereas experimentally, mice lacking Rec required approximately 9.7-fold more light to reach similar saturation times as WT (ref. [27]), the model required only approximately 1.6-fold more light to reach WT saturation times. Therefore, the model in fact fails to disentangle the effects of RK overabundance due to RK overexpression and Rec knockout mutations.

### Decreased RK availability slows recovery

**Figure 6:**
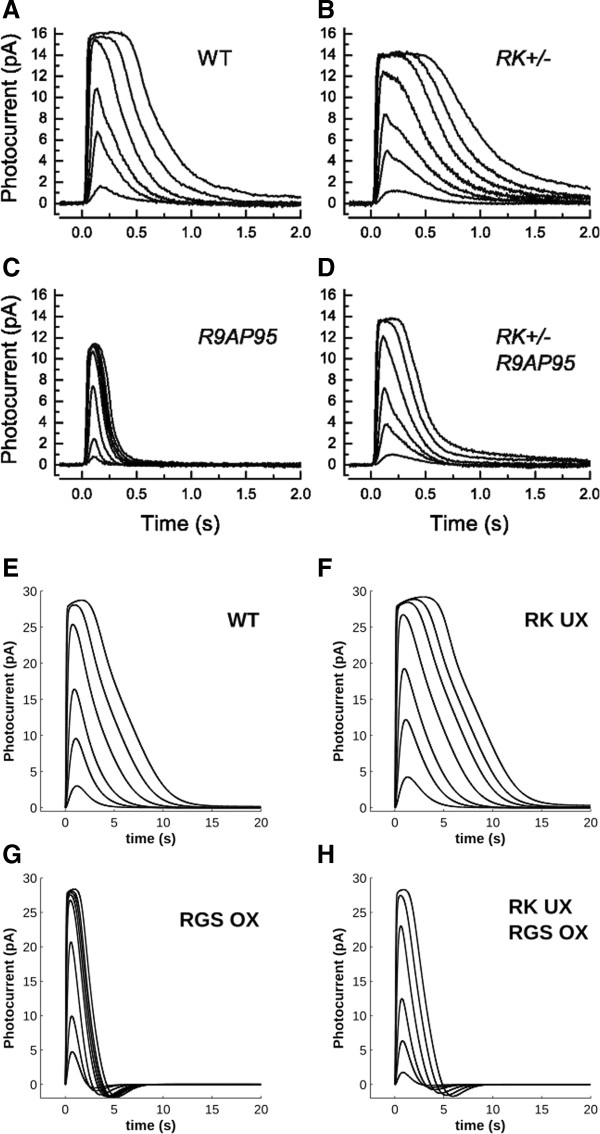
**Experimental (A-D; ref. **[28]) and simulated (E-H) flash responses showing that R* shutdown only becomes rate-limiting when RGS activity is greatly increased. When RK is underexpressed 0.4x against a WT background (**B**, **F**), the response shows a slight slowing in recovery time compared to WT (**A**, **E**). When RGS is overexpressed six-fold against a WT background (**C**, **G**), recovery is significantly accelerated. Finally, when RK is underexpressed against a background of RGS overexpression, the WT dynamics are partially recovered. Simulated stimulus intensities were adapted from ref. [28] (see Methods). WT (**E**) stimuli were 3.2, 13.6, 34.4, 127.2, 362.4 and 896 R* per flash. 0.4x RK underexpression (**F**) stimuli were 3.2, 13.6, 34.4, 127.2, 362.4, 690.4 and 1496.8 R* per flash. 6x RGS overexpression (**G**) stimuli were 13.6, 34.4, 127.2, 362.4, 516.8, 694.4, 896 and 1496.8 R* per flash. 0.4x RK UX/6x RGS OX double mutant stimuli were 3.2, 13.6, 34.4, 127.2, 362.4 and 896 R* per flash. (Panels **A**-**D** copyright © 2010 Chen et al.).

**Figure 7:**
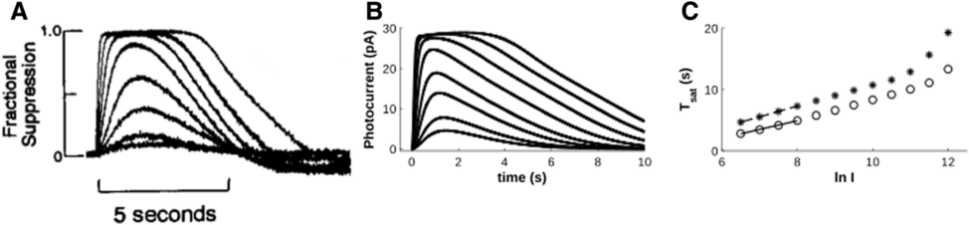
**Comparison between experimental (A; ref. **[29]**) and simulated (B) flash responses under conditions of Rec excess and a simulated Pepperberg plot (C) comparing these conditions (dashed lines, stars) to wild-type (solid line, open circles).** Rec excess was achieved by Gray-Keller *et al*. (ref. [29]) via rod outer segment dialysis of the protein and was simulated as 4x Rec overexpression. Stimuli were 3, 6, 15, 29, 70, 157, 317 and 570 R* per flash per ref. [29]. Rec overexpression results in a significant increase in saturation times compared to WT. (Panel **A** copyright © 1993 Cell Press).

Decreasing the expression level of RK allows one to directly test whether R* shutdown is the rate-limiting step for the recovery of the cascade. Moreover, measuring changes in T_sat_ and τ_D_ would allow one to assess quantitatively the effects over the whole illumination range. Indeed, it was found in transgenic mice which underexpress RK (0.4x) that the response dynamics are slowed significantly [24,28]. Reduced RK expression resulted in a slightly increased *τ*_*D*_ as well as a consistently increased *τ*_*rec*_. Similarly, when amphibian rod outer segments were dialyzed with an excess of Rec, response recovery was found to be significantly prolonged [29]. Simulating both of these cases resulted in response curves in agreement with the experimental data. For example, the recovery phase slowed down both for 40% underexpression of RK (compare Figure 6A and B) and for four-fold overexpression of Rec (compare Figure 7A and B). RK underexpression resulted in a negligible change in *τ*_*D*_ relative to WT, while Rec overexpression caused a large increase in this measurement (Table 1; note that the difference in timescale between simulated and experimental values arises from species differences).

To determine if R*-shutdown was indeed the rate-limiting process in rods underexpressing RK, Chen *et al*. produced double mutants that also overexpressed RGS (Figure 6C) [28]. If R*-shutdown were truly rate-limiting, then increased RGS activity should not have a significant effect on the response dynamics. However, it was found that the double mutants have faster shutdown dynamics relative to both WT and RK underexpressing rods (Figure 6D), though they are slower relative to rods which only overexpress RGS (Figure 6C). These results could be replicated in our simulations (Figure 6G and H) and further confirm that RGS-mediated effector shutdown is the rate-limiting step in phototransduction deactivation, as further demonstrated by the following simulations.

### Effector shutdown is rate-limiting

**Figure 8:**
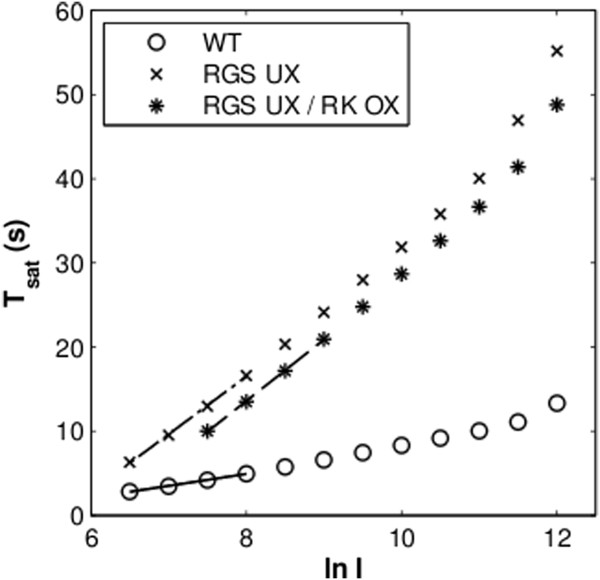
**Simulated comparison of Pepperberg plots for hypothetical 0.2x RGS underexpression animals (dotted-dashed lines, X’s), hypothetical double-mutants which also overexpress RK 3x (dashed lines, stars) and WT (solid line, open circles).** RGS underexpression results in significantly longer *T*_*sat*_ as well as steeper *τ*_*D*_. Additionally overexpressing RK has only a moderate effect on *T*_*sat*_.

According to current knowledge, RGS-mediated shutdown of the effector is the rate-limiting step of response recovery. The rate-limiting step is expected to primarily determine *τ*_*D*_, or in other words, how *T*_*sat*_ changes with increasing stimulus intensities. Thus, decreasing RGS activity is expected to greatly affect *T*_*sat*_ and *τ*_*D*_. Furthermore, since *τ*_*D*_ is ultimately affected by the rate-limiting step, complementing a scenario of lowered RGS activity with increased RK should not have any additional effect on *τ*_*D*_. In fact, when such a hypothetical experiment was simulated, we saw that the dominant time constants were approximately equivalent for rods that only underexpress RGS and double-mutants which additionally overexpress RK, (Table 1). However, we saw a consistent shift in *T*_*sat*_, for the double-mutant, which required approximately one half log step stronger stimuli to achieve the same *T*_*sat*,_as the single-mutant (Figure 8).

It has recently been reported that, as the key protein of the rate-limiting step, RGS’s expression level is extremely important to the recovery dynamics of the phototransduction system [30]. We took advantage of the model to verify these results. Despite the difference in species between this experiment and our model, the simulated results were qualitatively in agreement with the experimental data (Additional file 1: Figure S3). *τ*_*D*_ varies greatly with RGS expression levels in a manner consistent with the published experimental data. It should be noted that a simple model of RGS activity was included by Burns and Pugh to quantitatively explain RGS dynamics relying on Michaelis-Menten kinetics [30]. We show with the present model that we can arrive at the same conclusions by representing the system with only mass-action kinetics of the fundamental reactions involved therein (Additional file 1: Figure S3).

### Arrestin concentration does not affect recovery dynamics

**Figure 9:**
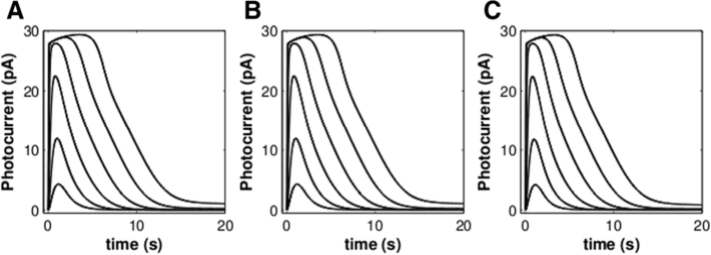
**Arr expression does not have a significant effect on the photoresponse. A)** WT **B)** 0.5x Arr underexpression, according to Gross and Burns (2010) [26]**C)** hypothetical 2x Arr overexpression. Flash stimuli were: 4.8, 19.2, 76.8, 307.2 1228.8 and 4915.2 R* per flash.

If Arr availability due to its oligomerization is important to recovery dynamics, then one might suppose that its concentration also may have a significant influence. However, Gross and Burns showed that underexpressing Arr (0.5x) in mice does not perturb the kinetics of photoresponses over a broad range of light stimuli (~5 - 5,000 R*/flash) [26]. We simulated both the 0.5x under- and 2x overexpression of Arr. In line with the experimental results [26], underexpressing Arr does not perturb the photoresponse (compare Figure 9A and 9B). Moreover, our model predicts that doubling Arr levels would also be ineffective (Figure 9C). Thus, it would appear that Arr’s affinity for itself works to maintain an ideal concentration of the monomeric form, even under conditions that would change its expression level.

### Arrestin dynamic oligomerization buffers increased RK activity

**Figure 10:**
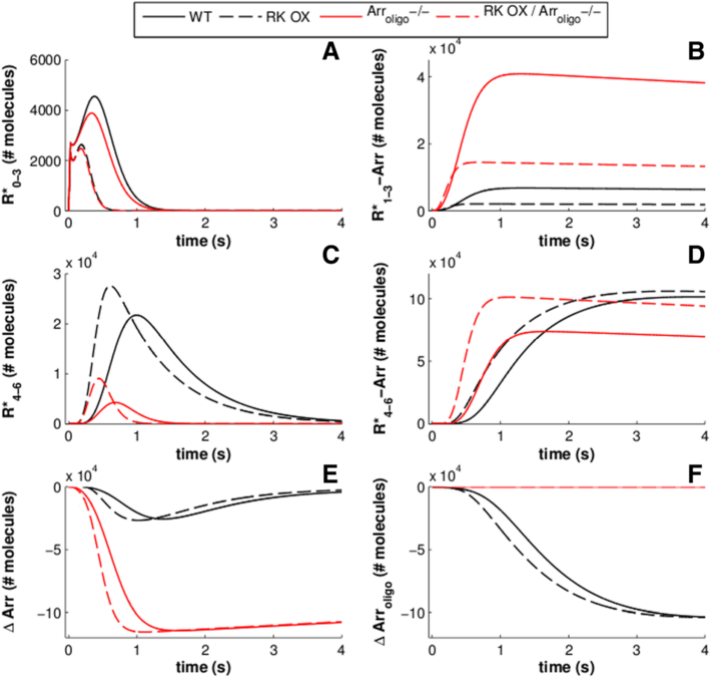
The evolution of the summed concentrations of several classes of molecular species during the first four seconds of a response to a saturating flash stimulus (118,000 R*). Black traces are simulated with normal Arr oligomerization while red traces are from simulations with Arr oligomerization disabled. Solid traces are simulated with normal RK expression while dashed traces are simulated with 2.4x RK overexpression. **A**) R* with zero to three phosphates attached. **B**) R* with zero to three phosphates and bound to Arr. Notice that when Arr cannot form homo-oligomers, it binds to sparsely phosphorylated R* more rapidly and at higher quantities. **C**) R* with four to six phosphates. When Arr oligomerization is disabled, fewer R* reach this state. **D**) R* with four to six phosphates and bound to Arr. When Arr oligomerization is disabled, heavily phosphorylated R* is bound by Arr quicker, an effect which is particularly influenced by RK expression levels. **E**) Monomeric Arr quantities begin to be depleted upon R* activation but they are quickly replenished by the oligomer stores. When Arr oligomerization is disabled, monomeric Arr quantities decrease by a much greater amount and do not recover. **F**) Arr molecules present in homo-oligomeric forms gradually decrease in quantity, replenishing the available monomer, and do not recover in quantity within the simulated time range.

A unique advantage of the model proposed here is that it allows the observation of the time-evolution of the concentrations of individual molecular species in the signaling network, thus revealing interconnections between the underlying dynamics. We traced the temporal evolution of selected molecular quantities, following a saturating flash stimulus leading to 118,000 R*, namely: i) R* with zero to three phosphorylations (Figure 10A); ii) R* with four to six phosphorylations (i.e. the maximum number in the model; Figure 10B); iii) R*-Arr complexes in which R* has been phosphorylated one to three times (Figure 10C); and R*-Arr complexes in which R* has been phosphorylated four to six times (Figure 10D). We also simulated the change in molecular quantities of Arr in monomeric and oligomeric form (Figure 10E and F, respectively). Results compared WT (black solid lines in Figure 10) and 2.4-fold RK overexpression conditions (black, dashed lines in Figure 10). Interestingly, it can be seen that most R* molecules under these conditions reach four to six phosphorylations before binding Arr. It should be noted, however, that it has been shown that three phosphorylation events are sufficient for monomeric Arr to bind R* (ref. [12]); indeed, we did find that some R* was bound to Arr with less than four phosphorylations (Figure 10B). When RK is overexpressed, more R* molecules unsurprisingly attain at least four phosphorylations (Figure 10C) and they are bound by Arr slightly more quickly (Figure 10D). Under both WT and RK overexpression conditions, Arr binding with less than four phosphorylations is completed within approximately one second (Figure 10B), while R* molecules with four or more phosphorylations require approximately three seconds to be completely bound by Arr (Figure 10D).

Observing the time evolution of Arr molecules in monomeric or oligomeric form supports the notion that Arr oligomers serve to maintain the amount of monomers that are available to bind with R*. After an initial decrease, the quantity of available Arr monomers quickly recovers to nearly the dark steady-state number (Figure 10E). The number of Arr molecules in an oligomeric form, on the other hand, steadily decreases and does not recover within the observed time frame, owing to the slow release of Arr from R* (Figure 10F).

We next investigated the kinetic effect of Arr oligomerization on R* shutdown by repeating the simulations with the rate of Arr self-association reduced to 0 s^-1^. With Arr oligomerization disabled, we found that a much larger percentage of R* binds Arr before four phosphorylations have been acquired (red traces, Figure 10A, B). Furthermore, Arr binding to R*, regardless of the level of R* phosphorylation, proceeds at a faster rate such that all R* is bound to Arr within approximately one second (red traces, Figure 10B, D). The rate is even faster under RK overexpression conditions. Thus, it is likely that Arr oligomerization is the primary mechanism that can account for the failure of the model of Dell’Orco *et al*. [15] to reproduce the lack of an effect of RK overexpression on the time spent in saturation after a bright stimulus.

## Discussion

The modeling approach used here differs from the classical approaches to modeling the phototransduction pathway by its focus on implementing each reaction in a fundamental form described by mass-action kinetics rather than via more abstract kinetics. By basing the model on the principal reactions, preferring linear combinations of parameters, realistic dynamics arise simply through the fundamental nature of the complex molecular interactions rather than through any introduced mathematical complexity. Thus, the model uses a “bottom-up” modeling approach, in which the process is understood through the emergent dynamics of a complex system, as opposed to a “top-down” approach, in which the observed dynamics are distilled down to mathematical relationships which can accurately reproduce them.

We have made significant steps forward in the comprehensive, biochemistry-based description of the phototransduction cascade, focusing on the still largely unclear mechanisms constituting limiting steps in the kinetics of cell recovery after illumination, by describing the Rec-RK interaction in the model with a representation quantitatively accounting for the experimentally determined kinetics and then by extending it to include Arr oligomerization (see Methods). The final model represents perhaps the most comprehensive molecular model of visual phototransduction to date.

Importantly, the new Rec-RK implementation did not significantly alter the dynamics of the model, while its simpler implementation compared to the previous version of the model allowed easier manipulation of these two proteins to clarify their own influence on the dynamics. Meanwhile, the novel Arr oligomerization mechanism allowed us to better understand the discrepancy between simulated and experimental responses of RK overexpression. Our simulated results confirm that the RGS-mediated effector shutdown is the rate-limiting step of phototransduction deactivation, as has been previously asserted. Furthermore, our approach allowed us to investigate this in finer detail than what may be feasible *in vitro* or *in vivo*.

We find that for the much faster process of R* shutdown, Arr is rate-limiting due to its homo-oligomerization. This has been alluded to in previous experiments which showed that saturation time after exposure to a bright stimulus is unaffected by an increase in RK activity due to its overexpression [23,24]. With our model, we could demonstrate that this effect is at least partially mediated by the kinetics of Arr oligomer dissociation. On the other hand, our model fails to capture the fundamental difference between an overabundance of available RK due to its overexpression and that due to the lack of Rec regulation of RK in Rec knock-out animals. This discrepancy merits further investigation.

Our simulated results also suggest that Arr oligomerization has a significant effect on the timing of R* shutdown and on phototransduction recovery dynamics in general, particularly at brighter stimulus intensities. While it ultimately serves to maintain sufficient concentrations of monomers available to shut down R*, the transition of Arr from its oligomeric storage forms to the monomeric form slows down availability for and ultimately its activity in the shutdown of the activated receptor. Thus, RK overexpression has no effect on saturation time because the rate of R* shutdown is ultimately determined by Arr availability. When Arr oligomerization is disabled, on the other hand, high concentrations of Arr are immediately available to shut down R* as soon as it is sufficiently phosphorylated by RK; when RK is overexpressed, this occurs at a faster rate and thus *T*_*sat*_ is reduced.

Because Arr’s three oligomeric states are expected to exist in equilibrium concentrations in the dark (ref. [31]), some monomeric Arr is always available to quench R* after a weak stimulus. In our model under dark conditions, approximately 13% of Arr is present in monomeric form (~3.8e7 monomers = ~63 μM), 27% is present in dimeric form (~4e7 dimers = ~67 μM) and 60% is present in tetrameric form (~4.5e7 tetramers = ~75 μM), given 3e8 total Arr molecules (Table 2). It is known that the dark steady-state concentration of monomeric Arr varies widely between species, implicating Arr’s affinity for itself as a primary evolutionary target for the tuning of the regulation of R* shutdown [31]. For example, while the formation of tetramers is highly cooperative in bovine photoreceptors, in mice the dissociation constants of dimer and tetramer formation are nearly the same. The resulting estimated concentrations of monomeric Arr in mice, cows and humans are 53 μM, 28 μM and 15 μM, respectively [32]. Thus, the dark concentrations of the three Arr forms that we find in our model have realistic values. However, because the dissociation constant of Arr self-association has not been measured in any amphibian species, the true values may vary from those estimated here.

**Table 2:** Steady-state protein concentrations

**Molecular species**	**# molecules**	**Concentration (μM)**
Arr	3.82e7	63.4
Arr_di_	4.04e7	67.1
Arr_tetra_	4.52e7	75.1
RK	8e3	0.01
RecR_Ca_ · RK	3.99e6	6.6
RecR_Ca_	1.87e6	3.1
RecT	1.41e7	23.4

Steady-state concentrations of Arr, RK and Rec in their various modeled states when no stimulus is presented. The model tracks molecular counts of each of these species. Equivalent micromolar concentrations were calculated using a rod photoreceptor outer segment volume of 1pL.

Finally, it is known that Arr undergoes translocation from the photoreceptor inner segment to the outer segment in a light-dependent manner [33]. This occurs in the time-span of minutes, much longer than the duration of experiments described herein, thus we should not expect to see its effect in such experiments [33,34]. Furthermore, many electrophysiological experiments, such as the experiments discussed here, use exclusively the rod outer segments, precluding any translocation. Nevertheless, the inclusion of Arr translocation in the model may result in a large portion of it being sequestered and completely unavailable during experiments on dark-adapted cells. As a result, the true kinetic parameters of Arr oligomerization may in fact be different than those that were herein estimated without translocation, depending on the time-course of the true concentrations of all Arr forms present in the outer segment during a photoresponse.

## Conclusions

We have demonstrated that the present model, particularly after the inclusion of Arr oligomerization, can accurately simulate many complex dynamics of visual phototransduction, even under mutant conditions, including simultaneous overexpression and down-regulation of genes involved in the regulation of the recovery kinetics. The advantages of such a model are clear: by breaking the process down into fundamental steps, largely modeled using mass-action kinetics, it is simple to realistically simulate the effects of perturbations to the system. Because the model is composed in a modular manner, adding new mechanisms or replacing existing mechanisms with more realistic representations based on new experimental data can be done without greatly disrupting the rest of the model. Because the model is largely comprehensive, it is possible to test the long-reaching effects of the complex interactions and feedback loops present in this system. It is hoped that this model can serve as both a useful tool in future research by providing mechanistic insights as well as a unified compendium of knowledge of the visual phototransduction process.

While the phototransduction dynamics simulated by the present model are largely accurate, there remain some aspects that deserve further attention. Most of the primary mechanisms known to occur during the phototransduction process have been included in the model. However some, such as light-induced translocation of Arr as described above, as well as that of Rec (ref. [35]) and G (ref. [34,36-39]), have yet to be integrated. Other processes, such as the action of phosducin or calmodulin, or the dynamic influence of the phosphorylation and dephosphorylation of phototransduction proteins other than R*, remain omitted from the model due to lack of mechanistic information. There remain processes which are present in the model in forms which assume dynamics other than those based on the law of mass action. For example, the mechanism of Ca^2+^-mediated activation of guanylate cyclase by GCAPs is not explicitly modeled. Improving its representation in the model would allow more flexible experimental manipulation of its parameters when probing its simulated dynamics.

Finally, we took advantage of the inherent modularity of the system to formulate and test the hypothesis that RK-mediated R* shutdown, particularly when RK is overexpressed, is modulated by Arr availability in its monomeric form. Integrating two new reactions into the model required relatively little compared to the time and costs involved in generating and maintaining transgenic or cross-bred animals. With the knowledge provided by the model, focused laboratory experiments to formally test this hypothesis can be undertaken with more confidence than had they been performed without the data provided herein. Indeed, this is one of the largest over-arching goals of the field of systems biology.

## Methods

### Model building

A previously published model of the phototransduction system was used as a template on which our changes were introduced [15], after first incorporating a R*-G pre-coupling mechanism described using the same model [19]. The network of reactions comprising the system was modeled in a deterministic manner, with each reaction being represented by an ordinary differential equation (for the full network of reactions see ref. [15]). Except where otherwise noted, the law of mass action was used to describe the kinetics. The change with time in the number of molecules of each chemical species was simulated by summing all of the reaction rates which produce the species and subtracting all of the rates which remove the species from the system. The model simulates the reactions taking place in a well-stirred volume, hence the spatial structure of the photoreceptor outer segment is not taken into account. The reactions building up the network were implemented as described in Additional file 1: Table S1 of Dell’Orco et al [15] and in Dell’Orco and Koch [19], and the parameters that needed tuning for the present implementation are reported in Table 3.

**Table 3:** Model parameter values

**Param.**	**Unit**	**Description**	**Reaction no.**	**Dell’Orco & Koch **[19]	**Present model**	**Present source**
kG1_0_	s^-1^	Rate of binding of Gt to unphosphorylated R*	DO-13	3.28e-5	2.416e-2	Optimized
*k*RK1_0_	s^-1^	Rate of binding of RK to unphosphorylated R*	DO-2	7.543e-3	5.198e-2	Optimized
*m*_*RK*_		Slope of the rate of decrease in RK affinity for R* with increasing phosphorylation events	DO-2		0.1	Manually tuned
*k*Arr	s^-1^	Rate of binding of Arr to R* with a single phosphate attached	DO-5	6.092e-10	6.204e-8	Optimized
*m*_*Arr*_		*Slope of t*The rate of increase of Arr-R* affinity with additional R* phosphorylations	DO-5		1.14e-8	Optimized
*k*A2	*s*^*-1*^	Rate constant of dissociation of R* from Arr · R* without R* deactivation	DO-5	3.232e-3	2.754e-4	Optimized
*k*A3	s^-1^	Rate constant of dissociation of R* from Arr · R* after R* deactivation	DO-6	4.451e-2	2.649e-2	Optimized
*k*A4	*s*^*-1*^	Rate constant of Arr self-association	3, 4		1.787e-8	Optimized
*k*A5	*s*^*-1*^	Rate constant of Arr self-dissociation	3, 4		0.646	Manually tuned
*k*RGS1	*s*^*-1*^	Rate of binding RGS to an effector complex	DO-22, DO-24	1.57e-7	1.86e-7	Optimized
*k*PDE_shutoff_	*s*^*-1*^	Rate constant of PDE-induced shutoff of an effector complex	DO-26, DO-27	3.3e-2	2e-2	Manually tuned
*k*Rec1	μM^-1^ s^-1^	Ca^2+−^dependent rate of Rec conformational change from “tense” to “relaxed” form	1		0.011	[43]
*k*Rec2	*s*^*-1*^	Rate of Rec conformational change from “relaxed” to “tense” form	1		0.05	[43]

Changes in parameter values relative to the model of Dell’Orco and Koch [19]. Reaction numbers prefixed with “DO-” refer to reaction numbers listed in the Additional file 1 of ref. [15]; otherwise they refer to reactions in the present work.

Before the described modifications were made to the model, the relationship between the level of R* phosphorylation and its affinity for other proteins was reconsidered. Previously, the affinity of R* for Gt and RK was modeled to decrease exponentially with each additional phosphorylation while the affinity of R* for Arr increased exponentially. Experimental evidence suggests that the affinity of R* for Gt does, indeed, decrease exponentially with additional phosphorylations [40]. The affinity of R* for Arr, on the other hand, has been shown to increase linearly with the first four phosphorylations [40,41]. In the model this relationship was changed from exponential to saturating linear. In accordance with experimental evidence, the affinity of Arr for R* was modeled to increase linearly for one-to-four phosphorylations and to remain constant for five or six. Thus, the on-rate was determined as

$${\mathrm{kA}1}_{n}=\left\{\begin{array}{ll} \mathrm{kArr}+m_{\mathrm{Arr}}*\left(n-1\right) & 1\leq n\leq 4\\ \mathrm{kArr}+m_{\mathrm{Arr}}*3 & n>4 \end{array}\right\}$$

where *n* is the number of phosphorylations, *k* Arr is the rate of binding of Arr to singly-phosphorylated R* and *m*_*Arr*_ is the rate of increase in affinity with each of the first four additional phosphates.

The effect of phosphorylation on the relationship between R* and RK is less well-understood. It has been suggested that the affinity of RK for R* depends both on its auto-phosphorylation as well as on that of R*, such that its affinity decreases with each phosphate added to either protein [42]. However, only the extreme cases of no phosphorylation and full phosphorylation were measured. We found that the model’s dynamics were strongly influenced by the previous exponential R*-RK relationship, particularly with regards to the RK overexpression experiments. We thus replaced it with a linear decrease in RK affinity for R* with increasing R* phosphorylation, which fit the model equally well. The rate of binding of RK to R* was defined as follows:

$${\mathrm{kRK}1}_{n}=\left\{\begin{array}{ll} {\mathrm{kRK}1}_{0}-m_{\mathrm{RK}}*n & 1\leq n\leq 5\\ 0 & n=6 \end{array}\right\}$$

where *m*_*RK*_ was set to *k*RK1_0_/5. Interestingly, it was found that setting the denominator of the slope to 5 led to the best fit, meaning that RK's affinity for R* is extinguished before R* reaches its maximum phosphorylation state.

The newly built model was tested alongside the previously published model, in a variety of light stimulation paradigms and under a variety of mutational scenarios. For example, the model reproduced the typical behavior of light adaptation in the presence of a non-saturating background light, consisting of reduced sensitivity and accelerated recovery (Additional file 1: Figure S1). When the signaling effector regulating protein RGS was knocked out, signal recovery was found to be drastically slower than normal, with perhaps more accurate kinetics than what was previously simulated (Additional file 1: Figure S2).

### Recoverin-RK interaction

Previously, Ca^2+^-mediated feedback on RK by Rec was modeled based on a quasi-steady-state assumption using a standard Hill equation to determine the quantity of Rec in the “relaxed”, Ca^2+^-bound form [14,15]. In order to more-accurately model the Ca^2+^-mediated feedback on RK by Rec, we replaced the original kinetics based on the quasi-steady-state assumption (Reaction 30 in ref. [15]) with a set of two fundamental reactions. First, Rec undergoes a conformational change upon binding Ca^2+^, from a “tense” form to a “relaxed” form with a covalently bound myristoyl group exposed (Figure 1):

(1) $$\begin{array}{l} \mathrm{RecT}+\left[{Ca}^{2+}\right]\overset{\mathrm{\upsilon f}}{\underset{\mathrm{\upsilon r}}{⇌}}{\mathrm{Rec}R}_{\mathrm{Ca}}\\ \;\mathrm{\upsilon f}=\mathrm{kRec}1\times \mathrm{RecT}\times \left[{Ca}_{\mathrm{free}}\right]\\ \;\mathrm{\upsilon r}=\mathrm{kRec}2\times {\mathrm{Rec}R}_{\mathrm{Ca}} \end{array}$$

To accommodate the difference in measurement in the model of Rec (number of molecules) and Ca^2+^ (micromolar concentration), when determining the rate of change of Ca^2+^ concentration the molecular quantities of RecT and RecR_Ca_ are dynamically converted to molar concentration using a volume of 1pL.

Dell’Orco *et al*. measured *k* Rec1 to have a value of approximately 0.011 μM^-1^ s^-1^ and *k*Rec2 was measured to be 0.05 s^-*1*^ using surface plasmon resonance [43], and these values were found to fit the data well. In the previous version of the model, the conformational change did not explicitly affect the Ca^2+^ concentration. Instead, the ion’s binding to Rec was included implicitly in its binding to a concentration of anonymous Ca^2+^ buffers (parameter *eT*). To account for Rec’s activity as an explicit buffer in the present model, the concentration *eT* was reduced by an appropriate amount.

Following the conformational change to the relaxed state, Rec is free to bind with RK:

(2) $$\begin{array}{l} {\mathrm{Rec}R}_{\mathrm{Ca}}+\mathrm{RK}\overset{\mathrm{\upsilon f}}{\underset{\mathrm{\upsilon r}}{⇌}}{\mathrm{Rec}R}_{\mathrm{Ca}}\cdot \mathrm{RK}\\ \;\mathrm{\upsilon f}=\mathrm{kRec}3\times {\mathrm{Rec}R}_{\mathrm{Ca}}\times \mathrm{RK}\\ \;\mathrm{\upsilon r}=\mathrm{kRec}4\times {\mathrm{Rec}R}_{\mathrm{Ca}}\cdot \mathrm{RK} \end{array}$$

*k*Rec3 and *k*Rec4 are analogous to the synonymous parameters in previous versions of the model. The values of *k*Rec3 and *k*Rec4 were held at the previously used values of 9.69 μM^-1^ s^-1^ and 0.61 s^-1^, respectively (Table 3), which had been attained by parameter estimation techniques. To accommodate the representation in the present model of Rec state in number of molecules rather than the previous representation in micro-molar concentration, the value of *k*Rec3 was converted into units of s^-1^, using an outer segment volume of 1pL. Because RK and Rec reach a steady state in the dark, the initial quantities of RecT, RecR_Ca_, RecR_Ca_ · RK, and RK were set at their steady-state values for all simulated experimental conditions (Table 2). We stress the fact that while minimal parameter tuning is necessary when new mechanisms are introduced in the model, the values of the optimized parameters were not subsequently changed in further simulations. Hence, all the predicted dynamic behaviors have to be considered as robust model validations.

### Arrestin oligomerization

The oligomerization of Arr was modeled according to mass-action kinetics in a simple two-reaction process. First, two Arr monomers bind reversibly to form an Arr dimer. Next, two dimers reversibly bind to form a tetramer. Thus, the first reaction is

(3) $$\begin{array}{l} \mathrm{Arr}+\mathrm{Arr}\overset{\mathrm{\upsilon f}}{\underset{\mathrm{\upsilon r}}{⇌}}{\mathrm{Arr}}_{\mathrm{di}}\\ \;\mathrm{\upsilon f}=\mathrm{kA}4\times {\mathrm{Arr}}^{2}\\ \;\mathrm{\upsilon r}=\mathrm{kA}5\times {\mathrm{Arr}}_{\mathrm{di}} \end{array}$$

while the second reaction is

(4) $$\begin{array}{l} {\mathrm{Arr}}_{\mathrm{di}}+{\mathrm{Arr}}_{\mathrm{di}}\overset{\mathrm{\upsilon f}}{\underset{\mathrm{\upsilon r}}{⇌}}{\mathrm{Arr}}_{\mathrm{tetra}}\\ \;\mathrm{\upsilon f}=\mathrm{kA}4\times {\mathrm{Arr}}_{\mathrm{di}}^{2}\\ \;\mathrm{\upsilon r}=\mathrm{kA}5\times {\mathrm{Arr}}_{\mathrm{tetra}} \end{array}$$

To minimize the number of introduced parameters, both reactions were assumed to occur at the same rates. Because the rates of these reactions have not been measured in amphibians, we chose to approximate them based on the known dissociation constants in mice, which approximately equal 60 μM for both reactions [32,44]. To this end, *k*A4 was estimated by parameter optimization techniques while *k*A5 was held fixed to maintain a K_D_ of 60 μM given an outer segment volume of 1pL (Table 3).

Since the concentration of Ca^2+^ changes dynamically upon illumination and the cation triggers regulation mechanisms throughout the cascade, we considered the possibility of a heretofore unknown mechanism of direct Ca^2+^ feedback on Arr oligomerization. This was implemented by adding an exponential dependence on free Ca^2+^ concentration as a fraction of the dark Ca^2+^ concentration for the two forward rates described above. For example, the forward rate of the first reaction would be

$$\mathrm{\upsilon f}=\mathrm{kA}4\times \exp\left({\mathrm{omega}}_{\mathrm{Arr}2}\times \frac{{\mathrm{Ca}}_{\mathrm{free}}^{2+}}{{\mathrm{Ca}}_{\mathrm{dark}}^{2+}}\right)$$

This extra mechanism did not impart any significant improvement on the fit of the model to experimental data. It was concluded that the indirect feedback of Ca^2+^ on Arr via Rec inhibition of RK was sufficient.

### Model implementation, parameter estimation and numerical simulations

Because of the novel mechanisms in the model, some signal recovery-related parameters from the original model required re-tuning. Parameter optimization was performed using local and global methods to minimize the change in quantitative response characteristics produced by the previous version of the model to 0.024 s flashes (1 R*/flash, 985 R*/flash, 15,600 R*/flash and 118,000 R*/flash) and 60s steps of light (48 R*s^-1^, 220 R*s^-1^, and 450 R*s^-1^). Local optimization was performed using the Nelder-Mead Simplex Method while global optimization was performed using a particle swarm method [45]. Parameter values were held fixed in all subsequent simulation experiments. Overall, the new model required moderate adjustments (Table 3). The Arr-related parameters required more significant re-tuning, which is not surprising given the change from an exponential dependence on R* phosphorylation state to a linear one and the addition of Arr oligomerization. The change in the relationship of RK affinity for R* and the phosphorylation state of R* from an exponential one to a linear one required significant re-tuning of the basal rate of binding of Gt to R* (*k*G1_0_). Because the affinity of RK for R* now decays at a linear rate, it continues to bind and phosphorylate R* when R* has more phosphates attached compared to the previous implementation; as a result, R* accumulates phosphates more quickly, resulting in a concomitant rapid decrease in the rate of Gt binding. To overcome this, the basal rate of Gt binding was required to be significantly faster. Note, however, that to the best of our knowledge, this rate has not been measured for amphibian Gt, thus the actual value is unknown. However, a faster interaction between Gt and R* is in line with recent surface-plasmon resonance determinations [19].

The model was implemented using SBTOOLBOX2 for Matlab (http://www.sbtoolbox2.org) [46]. SBTOOLBOX2 or SBML model files are available upon request. All numerical simulations were carried out in this framework, including parameter estimation. Deterministic simulations were run from automatically generated and compiled C-code models, based on the CVODE integrator from SUNDIALS [47].

### Model validation and experimental simulations

Simulated stimulus intensities were adapted from the published experimental procedures. Where experimental stimuli were measured in photons μm^-2^, they were converted to R* per flash using a collecting area of 0.4 μm^2^[48]. Where experiments were performed on mice, stimulus intensities were doubled, to qualitatively account for species differences in sensitivity between the model and the experimental animals.

To facilitate quantitative comparisons of *T*_*sat*_ and *τ*_*D*_ measurements between simulated experiments, we opted to standardize the methodology rather than simulate exactly the methodology of the published experiments. More specifically, whereas experimental procedures may define saturation time as the time spent above either 80% or 90% of the maximum current suppression, and *τ*_*D*_ may be calculated as the slope of either the first three or four points of the linear phase in a Pepperberg plot [25], we opted to exclusively measure currents above 90% of the maximum as saturating and to estimate *τ*_*D*_ from the first four data points on the curve. Furthermore, stimulus intensities for Pepperberg plots were standardized to half-log steps, with the same set of stimuli used for all such plots, regardless of those originally used in the published experiments.

## Abbreviations

Arr: Monomeric arrestin; Arrdi: Dimeric arrestin; Arrtetra: Tetrameric arrestin; G: Transducin; GCAPs: Guanylate Cyclase Activating Proteins; KO: Knock-out; OX: Overexpression; Rec: Recoverin; RecRCa: Ca^2+^-bound, “relaxed” recoverin; RecT: “tense” recoverin; RGS: Regulator of G-protein Signaling (protein); RK: Rhodopsin kinase; R*: Photoisomerized (activated) rhodopsin; Tsat: The length of time that a saturating photoresponse remains at greater than 90% of its maximum current suppression; τrec: The time constant of a single exponential function fit to the second half of the recovery phase of a non-saturating photoresponse; τD: The dominant time constant of recovery from a saturating response, measured as the slope of T_sat_ over logarithmically increasing stimulus intensities; UX: Underexpression; WT: Wild type.

## Competing interests

The authors declare that they have no competing interests.

## Authors’ contributions

BMI implemented the modifications to the model, carried out the experimental simulations and drafted the manuscript. LM assisted in the drafting of the manuscript. KWK assisted in the verification of the model. JB assisted in the drafting the manuscript. DDO conceived of the study, helped with the verification of the model and drafted the manuscript. All authors read and approved the final manuscript.

## Supplementary Material

Additional file 1: Figure S1 Simulated manifestations of light adaptation in WT rods illuminated by a saturating bright flash in the presence (dashed lines) or in the absence (solid lines) of a previous, nonsaturating steady illumination. Both models accurately reproduce the reduction in *Tsat* when exposed to a previous, steady illumination.This file contains supplementary figures demonstrating further experiments performed to verify the model which are mentioned but not reproduced in the main text. **Figure S2.** Simulated families of photoresponses from rods stimulated by flashes of increasing strength. While WT rods (**A**) recover normally, rods lacking RGS (**B**) show severely prolonged recovery. Note that the experimental and simulated time scales differ due to species differences, and that the experimental photocurrents are normalized to the maximum experimental photocurrent. Both models perform similarly. The present model features slower innate E* shutoff than previous models, thus they show slower recovery in RGS knockout experiments. **Figure S3.** Simulations of RGS expression experiments of Burns and Pugh (2009) [30]. τD, the rate of change in saturation time for increasing log stimulus intensities, is strongly dependent on RGS expression level (0.2x underexpression: X's; 2x overexpression: stars; 4x overexpression: squares; WT: circles). Click here for file
