# Supplementary material for: Exploring the rate-limiting steps in visual phototransduction recovery by bottom-up kinetic modeling
Source: Cell Commun Signal. 2013 May 21;11:36. doi: 10.1186/1478-811X-11-36 (PMC3732082; doi:10.1186/1478-811X-11-36)
Supplement: Additional file 1: Figure S1 — Simulated manifestations of light adaptation in WT rods illuminated by a saturating bright flash in the presence (dashed lines) or in the absence (solid lines) of a previous, nonsaturating steady illumination. Both models accurately reproduce the reduction in Tsat when exposed to a previous, steady illumination.This file contains supplementary figures demonstrating further experiments performed to verify the model which are mentioned but not reproduced in the main text. Figure S2. Simulated families of photoresponses from rods stimulated by flashes of increasing strength. While WT rods (A) recover normally, rods lacking RGS (B) show severely prolonged recovery. Note that the experimental and simulated time scales differ due to species differences, and that the experimental photocurrents are normalized to the maximum experimental photocurrent. Both models perform similarly. The present model features slower innate E* shutoff than previous models, thus they show slower recovery in RGS knockout experiments. Figure S3. Simulations of RGS expression experiments of Burns and Pugh (2009) [30]. τD, the rate of change in saturation time for increasing log stimulus intensities, is strongly dependent on RGS expression level (0.2x underexpression: X's; 2x overexpression: stars; 4x overexpression: squares; WT: circles). [file 1478-811X-11-36-S1.pdf]

Exploring the rate limiting steps in visual phototransduction recovery by bottom-up kinetic modeling

Brandon M. Invergo[1], Ludovica Montanucci[1], Karl-Wilhelm Koch[2], Jaume Bertranpetit[1], and Daniele Dell'Orco[3]*

1. IBE – Institute of Evolutionary Biology (UPF-CSIC), CEXS-UPF-PRBB, Barcelona, Catalonia, Spain

2. Department of Neurosciences, Biochemistry Group, University of Oldenburg, Germany

3. Department of Life Sciences and Reproduction, Section of Biological Chemistry and Center for BioMedical Computing (CBMC), University of Verona, Italy

**Corresponding Author:*

Daniele Dell'Orco
Department of Life Sciences and Reproduction
Sect. of Biological Chemistry and Center for BioMedical Computing (CBMC)
University of Verona
Strada le Grazie 8, 37134 Verona, Italy
Email: daniele.dellorco@univr.it
Phone: +39-045-802-7637
Fax: +39-045-8027170

Supplementary Figures

In the following two figures, we reproduce simulations performed by Dell'Orco *et al.* [1]. We compare the results using the model of Dell'Orco and Koch [2] (black traces) and the present model (red traces).

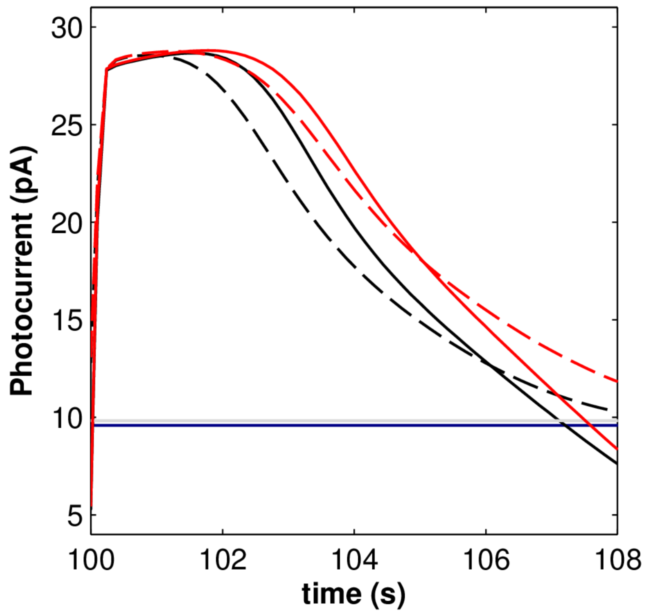

Figure S1: Simulated manifestations of light adaptation in WT rods illuminated by a saturating bright flash in the presence (dashed lines) or in the absence (solid lines) of a previous, non-saturating steady illumination. Both models accurately reproduce the reduction in T_{sat} when

exposed to a previous, steady illumination.

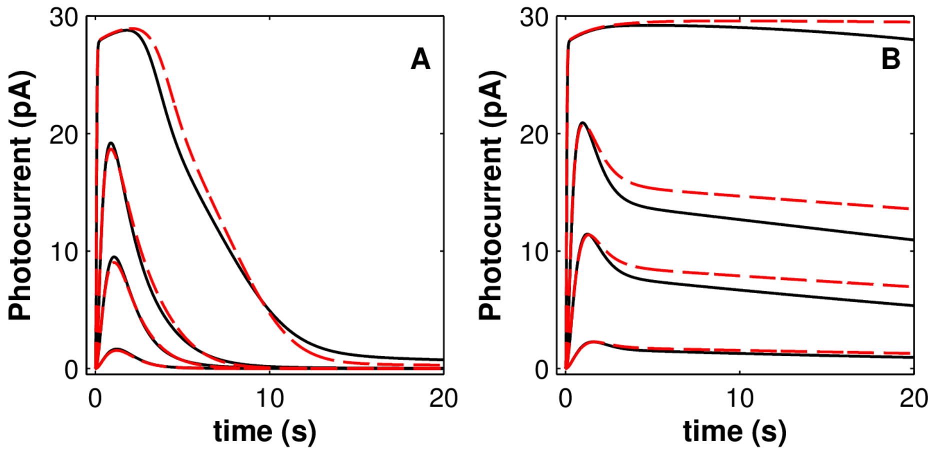

Figure S2: Simulated families of photoresponses from rods stimulated by flashes of increasing strength. While WT rods (A) recover normally, rods lacking RGS (B) show severely prolonged recovery. Note that the experimental and simulated time scales differ due to species differences, and that the experimental photocurrents are normalized to the maximum experimental photocurrent. Both models perform similarly. The present model features slower innate E* shutoff than previous models, thus they show slower recovery in RGS knockout experiments.

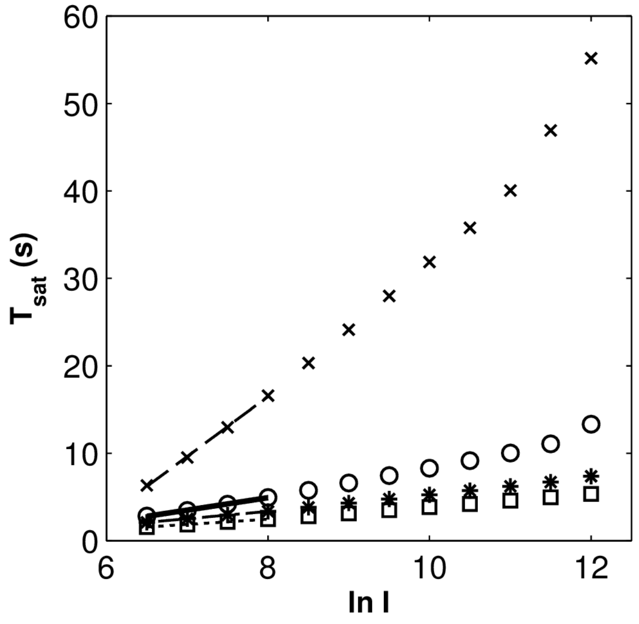

Figure S3: Simulations of RGS expression experiments of Burns and Pugh (2009) [3]. τ_D, the rate of change in saturation time for increasing log stimulus intensities, is strongly dependent on RGS expression level (0.2x underexpression: X's; 2x overexpression: stars; 4x overexpression: squares; WT: circles).

1. Dell'Orco D, Schmidt H, Mariani S, Fanelli F: **Network-level analysis of light adaptation in rod cells under normal and altered conditions**. *Molecular BioSystems* 2009, **5**:1232–1246.

2. Dell'Orco D, Koch K: **A dynamic scaffolding mechanism for rhodopsin and transducin interaction in vertebrate vision**. *Biochemical Journal* 2011, **440**:263–271.

3. Burns ME, Pugh EN: **RGS9 concentration matters in rod phototransduction**. *Biophysical Journal* 2009, **97**:1538–1547.
